# Deep transfer learning based image colorization using VGG19 and CLAHE

**DOI:** 10.1038/s41598-026-40292-1

**Published:** 2026-02-18

**Authors:** Neelanjan Ghosh, Gouranga Mandal

**Affiliations:** 1 Google LLC, Austin, TX USA; 2 Manipal Institute of Technology, Manipal Academy of Higher Education, Manipal, India

**Keywords:** Image colorization, Deep transfer learning, Pre-trained backbone network, Visual geometry group, CLAHE, Engineering, Mathematics and computing

## Abstract

Image colorization transforms grayscale images into realistic color representations. It is a challenging area of research in computer vision due to uncertainty in mapping intensity values to chromatic information. Earlier approaches, often based on optimization or reference-guided strategies, depend extensively on manual input or exemplary images. In contrast, recent advancements in deep learning methods enable more autonomous and reliable colorization. This article offers a deep transfer learning framework to accomplish high quality and efficient colorization of any gray-scale image. A pre-trained Visual Geometry Group (VGG19) network is developed as the backbone to extract both fine-grained textures and high-level semantic features. This network is designed with 16 convolutional & 3 fully connected layers. To get more prominent contrast and color in output image, the Contrast Limited Adaptive Histogram Equalization (CLAHE) is applied as a post-colorization step. CLAHE improves contrast and overall color quality of the output image. Experiments ware conducted on multiple datasets like ImageNet, COCO-Stuff and Places365. The experimental result proves that the proposed approach achieves outstanding performance in terms of PSNR and SSIM compared with prevailing techniques. Assessments of visual eminence also show improved color vibrancy and perfect contrast adjustment. The approach can be effectively used for restoring old photos, improving black and white movies, and enhancing black scanned medical images.

## Introduction

In computer vision, converting grayscale images into colorized versions remains a crucial and challenging task, mainly because a single intensity value might represent a wide range of useful color options. Enriching training data for different machine learning tasks, improving archive images, film material, and medical imaging are just a few of the many uses for the capacity to generate true colors.

Earlier approaches of image colorization mostly relied on manual intervention or direction based on references. Through the use of smoothness restrictions, users were able to apply color suggestions or “scribbles” to the image using optimization-driven techniques like those first presented by Levin et al.^1^. Similarly, via blurring local or global feature matching, exemplar-driven approaches transferred color information from reference images to grayscale inputs^2^. These approaches produced realistic results, but they were less dependable when the inputs were vague, sparse, or contextually incompatible because they mostly depended on thorough human annotations or well selected reference photos.

Automatic image colorization has advanced greatly since the advent of convolutional neural networks (CNNs). A CNN-based system that converts luminance values into chrominance components within the CIE Lab color space was proposed by Zhang et al.^3^, demonstrating a high capacity to capture semantic information. Building on this, Iizuka et al.^4^ created a dual-pathway architecture that enhances color transform accuracy by combining fine-grained local information with global scene context. Later, by learning to generate more varied and natural color distributions, generative adversarial networks (GANs)^5^ significantly improved visual sanity.

Even with these advancements, CNN-based model training from scratch requires large datasets and a lot of processing power. A solution is provided by deep transfer learning, which can reduce training weights while extracting powerful semantic and textural information for colorization by utilizing pre-trained models on massive datasets like ImageNet. For example, combining the perceptual loss functions with pre-trained backbones such as VGG or ResNet topologies improves performance^6,7^.

**Fig. 1:**
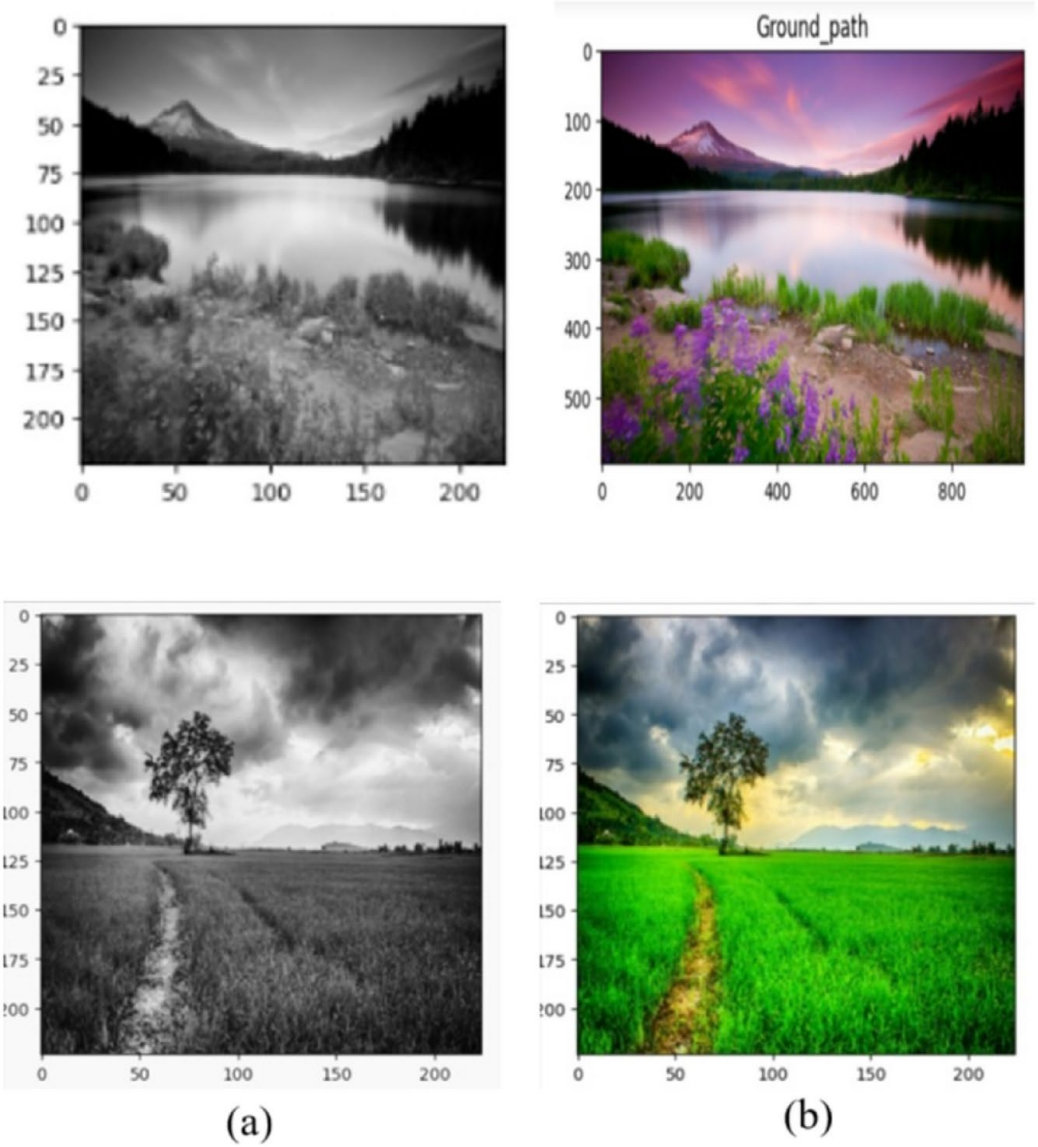
Grayscale image and its color ground truth, (**a**) grayscale image, (**b**) Color ground truth.

It is observed that most of the existing methods produced output images which are not as natural as the ground truth. A few methods somehow produced an almost natural color image, but the contrast is not prominent, which degrades the quality of the output image. The primary motivation of this research is to a produce perfect natural color image with optimal contrast balance from any gray-scale input image using deep transfer learning with adaptive contrast enhancement. A two sample gray-scale image and its color transformation are shown in Fig. 1.

The strategic contributions of this article are as follows:

- We propose a CNN-based colorization framework that leverages deep transfer learning, using a pretrained backbone (VGG19) for semantic and texture feature extraction.
- Amalgamation of Contrast Limited Adaptive Histogram Equalization (CLAHE) acts as a post-processing step, producing very prominent color output image with perfect contrast adjustment.
- We conduct extensive experiments on benchmark datasets, demonstrating marked improvements over baseline models in PSNR, SSIM, and FID metrics.
- Unlike traditional models, our approach effectively balances low-level detail and high-level semantics by aggregating features across multiple backbone layers.
- The method generalizes well across domains, making it well-suited for various traditional tasks such as colorizing historical gray photos, colorization of medical scan images, etc.

This is how the rest of the paper is arranged: The work in guided, automatic, and transfer learning-based colorization is reviewed in Section “Literature review”. Our suggested architecture and strategy are described in Section “Proposed methodology”. The experimental configuration and output results are demonstrated in Section “Experimental results”. Limitations of the proposed method are discussed in Section “Limitation”. Finally, the conclusion and outline of future directions are described in Section “Conclusion and future scope”.

## Literature review

Research in image colorization has undergone rapid evolution in the past five years (2020–2025), driven by deep learning, transformers, and human–AI collaboration. The literature study can broadly be grouped into three categories: (A) Hybrid Exemplar and Scribble-Based Methods, (B) Automatic Deep Learning Approaches, and (C) Transfer Learning and Efficient Architectures.

### Hybrid exemplar and scribble-based methods

Hybrid methods incorporate both automated learning and human input in the form of exemplary or scribbles. These existing methods are suitable for all types of black and white images in various fields.

Xiao et al.^8^ established a cooperative deep colorization framework where nominal user doodles are broadcast to produce natural-colored photos. This method can effectively preserve the semantic consistency of an image during transformation from grayscale to a colored version and make it precise. Furthermore, Yuan et al.^9^ introduced an exemplary-conditioned attention framework. This framework can transform colors from a grayscale image input. In contrast to earlier exemplary-based approaches, this model handled semantic inconsistencies very effectively. It applies attention mechanisms across both global and local scene data.

Lee et al.^10^ familiarized a hybrid approach that combines user input with a pre trained features derived from large-scale vision models. Their procedure maps user-specified hints into a semantic feature space, which improves steadiness and decreases artifacts of color bleeding in various scenarios. Approaches of this kind mainly focus on balancing between manual controls and automated processing. This method is very useful for professional artists and heritage restoration specialists, where independent preference and accuracy are crucial.

Overall, the evolution of study in this area signifies a shift toward fully automated colorization, reducing the necessity for direct user involvement. However, interactive methods that incorporate user hints or exemplar references continue to play an important role in niche domains, where precise control and domain-specific preferences are essential.

### Automatic deep learning approaches

Automatic deep learning–based models aim to eliminate user interaction by learning a transformation from grayscale to color image using large-scale datasets. These methods have rapidly progressed from CNN-based systems to GANs and, more recently, transformers.

Su et al.^11^ introduced contextual loss into CNN-based frameworks, arguing that conventional pixel-level losses often lead to desaturation. Their contextual loss improved semantic fidelity, producing visually pleasing results on natural scenes. Patel et al.^12^ advanced the field by using a GAN-based model enhanced with a style loss term, which apprehended global artistic styles and reduced the common “washed-out” effect seen in the prior CNN methods.

A significant revolution came with transformer-based models. Bahng et al.^13^ established a colorization transformer capable of modeling long-range dependencies called ColTran that captures local context. It empowered globally comprehensible color assignments and significantly beat the GANs on benchmark datasets. Kim et al.^14^ advanced this research work by adding a GAN–transformer-based hybrid model that combines the fine-grained sharpness achieved by GANs with the global contextual thoughtfulness of transformers. It carried enhancements in both perceptual practicality and operational accuracy.

Collectively, these contributions touch the evolution of colorization techniques—from CNN-based frameworks to GANs and, more recently, transformer-driven architectures, highlighting a steady drive toward additional coherent and visually conclusive automatic colorization systems.

### Transfer learning and efficient architectures

Although automatic approaches have demonstrated outstanding progress, their substantial computational demands pose several challenges during real-time implementation. For the last five years, the research focused on adopting transfer learning and model efficiency for scalable solutions.

Royer et al.^15^ verified that downstream colorization tasks, particularly in low-data regimes can significantly improve performance by using self-supervised pretraining on large-scale unlabeled datasets. Their results emphasized the role of pretraining as a substitute for labor-intensive dataset curation. Similarly, Zhang et al.^16^ proposed a ResNet-based transfer learning framework that unites perceptual loss, enabling faster convergence and better semantic alignment across various datasets.

Building on this, Liu et al.^17^ proposed a multi-scale VGG feature fusion strategy. By incorporating features from different layers of pretrained networks, their model preserved both global context and local details, resulting in improved sharpness and fewer artifacts. Rao et al.^18^ addressed the difficulties of deploying heavy GANs on mobile by presenting a model distillation method.

### Enhancement-based methods

The real-time colorization obtained good results while reducing the number of parameters by more than 60%. It is suitable for consumer-based color enhancement applications. The attenuated channel transfer based color enhancement is used for colorization^19,20^.

This evolution replicates a movement from experimental prototypes toward applied applications. Here, efficiency, scalability, and accessibility are considered crucial factors^21^. Patel et al.^12^ used a GAN-based model enhanced with a style loss term, which apprehended global artistic styles and reduced the common “washed-out” effect seen in the prior CNN methods. Besides, there are multiple low light image enhancement methods^22,23^.

In summary, the research field of image colorization has progressed from interactive, user-guided strategies to modern deep learning models incorporating CNNs, GANs, and transfer learning. While guided methods remain constrained, they depends on manual input. Automated approaches have demonstrated greater scalability and semantic reliability. Transfer learning improves generalization across datasets and lowers the overall training burden. Despite these advances, persistent problems such as dataset bias, heavy computational requirements, and difficulties in rendering fine-grained textures remain present. These limitations motivate the development of our CNN-based transfer learning framework, designed to deliver accurate, efficient, and visually coherent colorization. A comparative analysis of state-of-the-art approaches is provided in Table 1.

**Table 1:** Comparison of recent image colorization approaches.

Year	Approaches	Dataset used	Strengths	Limitations
2020	User-guided CNN^8^	Grayscale Face Dataset^8^	High local accuracy	Requires user input
2020–2021	GAN-based Auto Colorization^9^, Attention-guided CNN^11^	CIFAR-10^9^, CelebA-HQ^11^	Realistic textures, preserves details	High computational cost, slow training
2021–2022	Transfer Learning with VGG^10^, Self-supervised Pretraining^13^	ImageNet^10,24^, Places^13,25^	Faster convergence, reduced labeling cost	Limited generalization, weaker on small datasets
2022	Transformer-based Colorization^12^	DIV2K^12^	Captures global context	Memory-intensive
2023	Multiscale CNN^14^, Knowledge Distillation^15^	BSD500^14^, CIFAR-100^15^	Strong edge preservation, lightweight models	Blur in complex regions, slight accuracy drop
2024	CLIP-based Semantic Guidance^16^, Diffusion Models^17^	LAION-400M^16^, FFHQ^17^	Strong semantic alignment, high fidelity	Resource-heavy, slow inference
2025	Transfer Learning with Vision Transformer^18^, attenuated channel transfer^20,21^	ImageNet-21k^18^	Superior generalization	Needs large-scale GPU clusters

## Proposed methodology

The proposed methodology integrates preprocessing, transfer learning, CNN-based colorization, and post-processing in a multi-dataset learning framework. To achieve strong generalization, five benchmark datasets are utilized: ImageNet (available at: https://www.kaggle.com/datasets/dimensi0n/imagenet-256)^24^, Places365 (available at: https://www.kaggle.com/datasets/benjaminkz/places365)^25^, CIFAR-100 (available at: https://www.kaggle.com/datasets/fedesoriano/cifar100)^26^, CelebA (available at: https://www.kaggle.com/datasets/jessicali9530/celeba-dataset)^27^, and COCO-Stuff (available at: https://www.kaggle.com/datasets/phannguynhuphong/coco-stuff)^28^. Each dataset contributes distinct semantic strengths—ImageNet^24^ provides object-level diversity, Places365^25^ captures scene-level understanding, CIFAR-100^26^ emphasizes small-object variations, CelebA^27^ ensures facial fidelity, and COCO-Stuff^28^ enhances contextual scene representation.

### Preprocessing

**Fig. 2:**
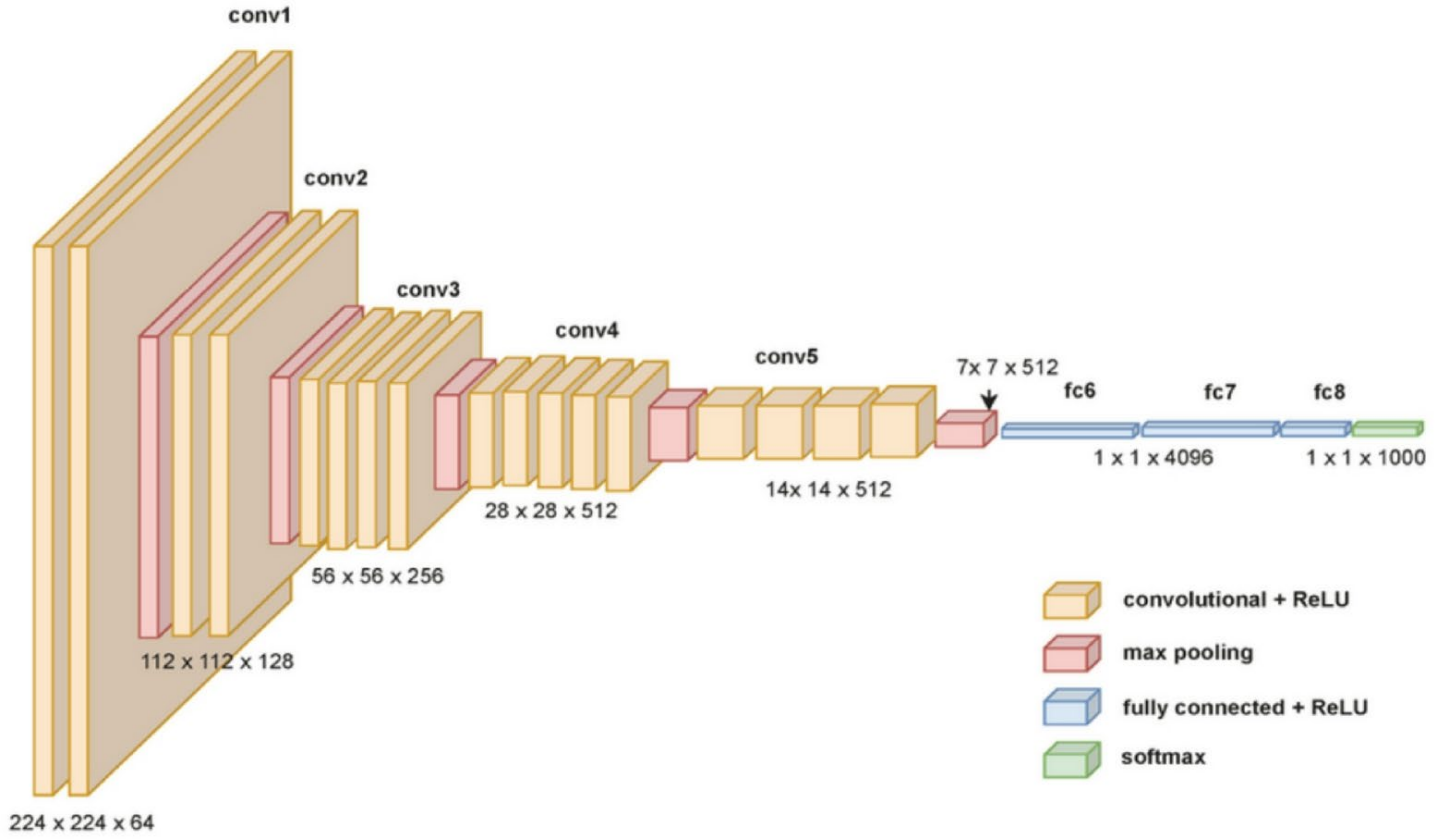
Overall model architecture of VGG19.

All RGB images are transformed to the CIELab color space during the preprocessing step, where the chrominance (a, b) channels must be predicted and the luminance (L) channel is used as input. Depending on the features of the dataset, photos are shrunk to a consistent scale. For example, bigger natural photographs are standardized to 256 × 256 pixels, while smaller datasets, like CIFAR-100^26^, maintain resolution appropriate for fine-grained details. To increase diversity and resilience, augmentation techniques including rotation, random cropping, and horizontal flipping are used sparingly. In order to stabilize convergence during training, all pixel intensities are normalized to the range of − 1 to 1^29^. Figure 2 illustrates the overall neural-based model architecture of VGG19.

### Transfer learning for feature extraction

To enhance semantic feature extraction, a transfer learning model is introduced. Pretrained models such as ResNet-50 and ResNet-101 provide robust object and scene-level priors, whereas VGG-based networks are fine-tuned for specific domains like facial attributes. This method allows the model to leverage earlier acquired visual features. It reduces training time and improves overall colorization accuracy. By employing dataset-specific transfer strategies, the network model can better align its feature illustrations with the semantic characteristics of the input images. The core part of this approach is transfer learning. It uses previously learned models that are trained on very large-scale datasets and applies them to a less labeled dataset for some new problems. Accumulating wide annotated colorization datasets is very rare and difficult. That’s why transfer learning is extremely beneficial in certain circumstances. The methodology starts with selecting an appropriate pre-trained CNN model, such as VGG or ResNet, and modifies it by adding layers accordingly to predict color information from grayscale inputs. A carefully curated dataset of grayscale images is considered for training with their corresponding color images. During the preliminary training stages, the pre-trained layers are frozen to reserve the learned visual representations, while the newly added layers are augmented for the colorization task.

### CNN-based colorization network

The proposed colorization framework employs an encoder–decoder CNN architecture. The encoder extracts both global contextual information and local spatial features, while an attention-based fusion module emphasizes significant structures, including object edges, scene layouts, and facial regions. The decoder then reconstructs the chrominance channels through transposed convolutions, and skip connections are incorporated to retain fine-grained spatial details, mitigating information loss across network layers. This architecture ensures that the system adapts to diverse image domains, producing contextually coherent and visually realistic colorizations.

### Training strategy

The training is performed in three stages. First, generic pretraining is conducted using large-scale datasets to establish general object and scene representations. In the second phase, fine-tuning is carried out on more specialized domains, such as small objects, enhancing detail. Finally, joint optimization across all datasets produces a robust, domain-generalized model. The learning process is adjusted using Adam from a preliminary learning rate of 1 × 10^−4^, scheduled dynamically using cosine annealing. The loss function combines pixel-wise mean squared error (MSE) with perceptual loss from VGG features^30^, while adversarial loss^31^ further improves realism in generated colors.

### Hypercolumn representation

**Fig. 3:**
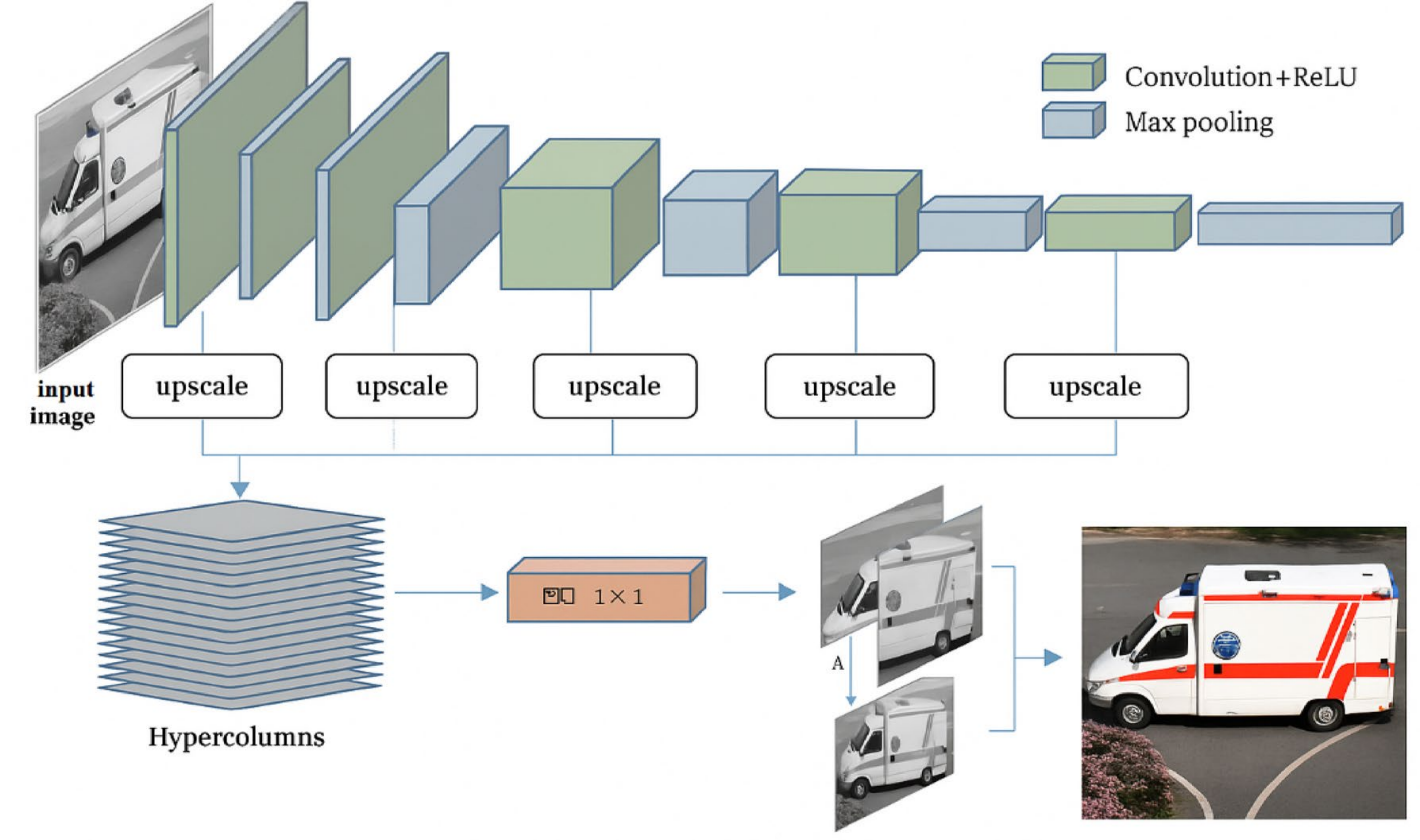
Detail framework of VGG19 deep-convolutional architecture. After adjusting the input image scale to create a hypercolumn layer, feature mappings are combined into layers 1 through 5 of the VGG19 network. The color image is created by combining the color channels A and B from the hypercolumns from gray image using a Conv 1 × 1.

Although the middle levels of a CNN are typically less semantically sensitive, the information acquired in the higher layers is also present in the intermediate layers. Hariharan et al.^32^ introduced the layers of a convolutional network as a non-linear analogue of picture pyramids. It is naturally utilized in optical flow and other vision-related applications of moving object detection and various image segmentation. Their method is predicated on the idea that pertinent data is dispersed among several CNN layers and ought to be utilized in concert. A “hypercolumn” is defined as a chain of outputs from all units above a specific input location across a subset of the network’s layers (practical implementations often sample only a subset of layers due to strong correlations between neighboring layers). They introduce this concept at a specific input location. The VGG19 architecture employed in this work is shown in Fig. 3. V1 neurons that are sensitive to edges at different alignments and frequencies are referred to as "hypercolumns," a word drawn from neuroscience. Here, it is expanded to provide a more thorough feature representation by using both higher-level semantic units and low-level edge detectors.

For each pixel, features are obtained by collecting the outputs of units from multiple layers corresponding to that pixel’s location. While each CNN layer produces feature maps, subsampling and pooling operations cause these maps to differ in spatial resolution from the original input, making it unclear which units correspond to a given pixel. To address this, the feature maps are resized to match the input image dimensions using bilinear interpolation. The features from all or selected layers are then concatenated to form a single vector for each pixel, referred to as a hypercolumn.

There are various color models with three basic color channels, including RGB, Lab, HSL, and HSV, by which any color image can be represented. In the colorization task, it is crucial to preserve the actual luminance information of the original grayscale image while predicting the color components. The RGB space exhibits strong inter-channel correlations, meaning that changes to color components often affect image brightness, which is undesirable. To minimize such interference, a color space that reduces correlation between luminance and chrominance is preferred. The Lab color space is particularly suitable, representing images with three values: luminance (denoted by *L*), green–red axis (denoted by *a*), and blue–yellow axis (denoted by *b*). Lab covers the full range of colors visible to the human eye and has minimal correlation among its channels, making it ideal for colorization. In this work, the *L* luminance channel is taken as the input grayscale image, while the network predicts the matching “a” and “b” color channels.

### Post-processing enhancement

After initial colorization, results are refined through Contrast Limited Adaptive Histogram-Equalization (CLAHE) is applied to the luminance channel to get prominent color and contrast adjustment. This technique improves local contrast, enhances edge sharpness, and balances tonal variations, producing visually appealing outputs across objects, scenes, and faces. The addition of this step ensures that the reconstructed images achieve both semantic fidelity and perceptual quality^33,34^.

A unique color level range is defined as follows, where L is 255 for an 8-bit frame, and each pixel’s color intensity is denoted by *i*. Here, a color level of minimal saturation tends to 0. But a range of *L–1* stipulates an extreme level of saturation. The *T(i)* function indicates the color levels*,* which determine the assignment of a new color value to pixels. Explicitly, *T(i)* function counts how many times a pixel is allotted to a color level other than its previous color. It allows the system to monitor color distribution throughout all pixels of the image.

1 $$s = T(i), \, 0 \le i \le L - 1$$

The color levels of an image can be represented by a random variable which ranges between 0 and L-1. The cumulative distribution function (CDF) represents the probability that a variable takes on a value within a specified range. Using a normalized histogram, the cumulative distribution for an image can be expressed as follows:

2 $$cdf(i \le t) = \sum\limits_{k = 0}^{t} {\mathop p\nolimits_{k} }$$

Equation (2) expresses the cumulative distribution function (CDF), which indicates the prospect that the color level of a given pixel is less than or equal to a specific value. Accordingly, the following definition can be used to describe the probability of seeing a particular color level at any random pixel:

3 $$\mathop p\nolimits_{k} = \frac{{\text{amount of pixels with color level k}}}{{\text{total number of pixels}}}$$

The probability that a randomly selected pixel has a color level of *k* is represented by the symbol *p*_*k*_. Following Eq. (2), the transformation step defines the function *T(i)* as follows:

4 $$\mathop s\nolimits_{k} = T(i) = floor\left( {(L - 1) * \sum\limits_{k = 0}^{i} {\mathop p\nolimits_{k} } } \right)$$

In this context, the floor () function is applied to round fractional values to the nearest integer. A simplified form of Eq. (4) is presented below^33,34^.

5 $$S_{k} = (cdf(i \le t) \times (L - 1))$$

Using this approach, the color levels are adjusted across all three channels, resulting in an equal distribution throughout the entire image frame.

## Experimental results

A PC with an Intel Core i7 13th Gen 1355U processor, 16 GB of RAM, 512 GB of SSD, and a GeForce GTX 1080 GPU with 8 GB of RAM made up the experimental setup. Since the model was evaluated on various datasets from Places365^25^, ImageNet^24^, and COCO-Stuff^28^ and trained on the extensive COCO-Stuff^28^ dataset, cross-validation is not used in this work. Specifically designed for object detection, picture segmentation, and captioning, the COCO-Stuff dataset^28^ is a subset of COCO^28^ that comprises around 118,000 training and 5,000 validation images organized into 172 classes (80 “things,” 91 “stuff,” and one unlabeled class). Before training, every image was resized to 224 × 224 pixels. The Places365 dataset is used to train pre-trained weights from the VGG16 model for scene ground-truth. After calculating the scene possibilities for 5,000 validation photos from COCO-Stuff^28^, the top five probabilities were selected, and the results were normalized so that they added up to 1. The first 1000 photos from the ImageNet, Places365, and COCO-Stuff^28^ validation sets, as well as 100 images from the high-resolution validation set, were used for colorization evaluation. The code is made publicly available at the following link https://github.com/gourangamm/Image-Colorization-Using-VGG19-and-CLAHE.git

For the performance assessment of the proposed framework, two types of metrics were employed: quantitative measures and perceptual quality metrics.

### Quantitative performance evaluation

The quantitative evaluation metrics were categorized into two groups: similarity-based measures and perceptual approaches. For the similarity group, this study employed two extensively used metrics: Peak Signal-to-Noise Ratio (PSNR) and Structural Similarity Index (SSIM)^34,35^. PSNR is the measure of pixel-wise fidelity between an enhanced image and its original reference. Normally, the reference image is considered noiseless, and PSNR calculates the change after enhancement. It is calculated in Eq. 6, where MSE is the mean square error of pixel intensities.

6 $${\mathrm{PSNR}}=10\mathrm{log}10\frac{{255}^{2}}{MSE}$$

The general interpretation of PSNR is ‘the higher the value the lesser enhancement’, with the range being 10–50. Since smoke removal modifies contrast and luminance, PSNR typically decreases slightly, but moderate PSNR values indicate structural consistency.

SSIM measures perceptual similarity between two images based on luminance, contrast, and structure. It is calculated using Eq. 7, where µx, µy are the mean intensity of original and enhanced images, σx, σy are the standard deviation in contrast, σxy is the covariance between images (structural similarity), and C1, C2 are constants for stabilizing division.

7 $$SSIM(x,y) = \frac{{(2\mu_{x} \mu_{y} + C_{1} )(2\sigma_{xy} + C_{2} )}}{{(\mu_{x}^{2} + \mu_{y}^{2} + C_{1} )(\sigma_{x}^{2} + \sigma_{y}^{2} + C_{2} )}}$$

The range for SSIM is 0–1, with 0 being no similarity and 1being the same image.

For color images, these metrics were calculated for each channel, and the average value was reported. PSNR and SSIM were evaluated on output-colored images, providing measures of reconstruction fidelity and structural similarity between the ground truth and the colorized outputs. However, these no-reference similarity metrics capture perceptual quality effectively. This perceptual metric was employed, which evaluates the alignment of metric scores with human visual perception under common distortions, such as noise, photometric variations, blur, geometric warping, and compression. This is achieved by computing the cosine distance between normalized feature vectors extracted from the images.

For evaluating quality metrics, we relied on visual inspection to highlight both successful and unsuccessful colorization cases for comparison. Visual performance was assessed using images from the ImageNet^24^, COCO-Stuff^28^, and Places365^25^ datasets. For testing, the first 500 images from the validation sets of each dataset were selected to evaluate the colorization results.

Table 2 presents the quantitative comparison results based on similarity metrics. For both PSNR and SSIM, higher values signify better performance. The User-guided CNN^8^ method and GAN-based Auto Colorization^9^ gave very good PSNR and SSIM results on ImageNet dataset especially on Place365 dataset. Transfer Learning with Vision Transformer^18^ also produced very good results on all three datasets. However, our proposed combined methods provided the best overall results on all the datasets. The results indicate that integrating CLAHE with the VGG19 architecture significantly contributed to improving colorization performance, particularly enhancing the accuracy of the predicted “a” and “b” color channels.

**Table 2:** Comparison of quantitative performance metrics of various methods on diffrent datasets.

Dataset	ImageNet		COCO-Stuff		Places365	
Method	PSNR	SSIM	PSNR	SSIM	PSNR	SSIM
User-guided CNN^8^	22.841	0.868	22.893	0.797	25.372	0.745
GAN-based Auto Colorization^9^	22.585	0.878	23.91	0.799	25.09	0.749
Transfer Learning with VGG^10^	21.297	0.848	21.88	0.777	23.07	0.732
Transformer-based Colorization^12^	22.002	0.859	22.231	0.792	23.595	0.748
Multiscale CNN^14^	21.768	0.869	21.694	0.797	22.91	0.742
CLIP-based Semantic Guidance^16^	21.09	0.839	22.33	0.797	23.858	0.751
Transfer Learning with Vision Transformer^18^	22.94	0.851	22.81	0.781	23.995	0.758
**Proposed Method with VGG19 + CLAHE**	**23.12**	**0.881**	**23.24**	**0.801**	**26.267**	**0.798**

### Qualitative performance evaluation

**Fig. 4:**
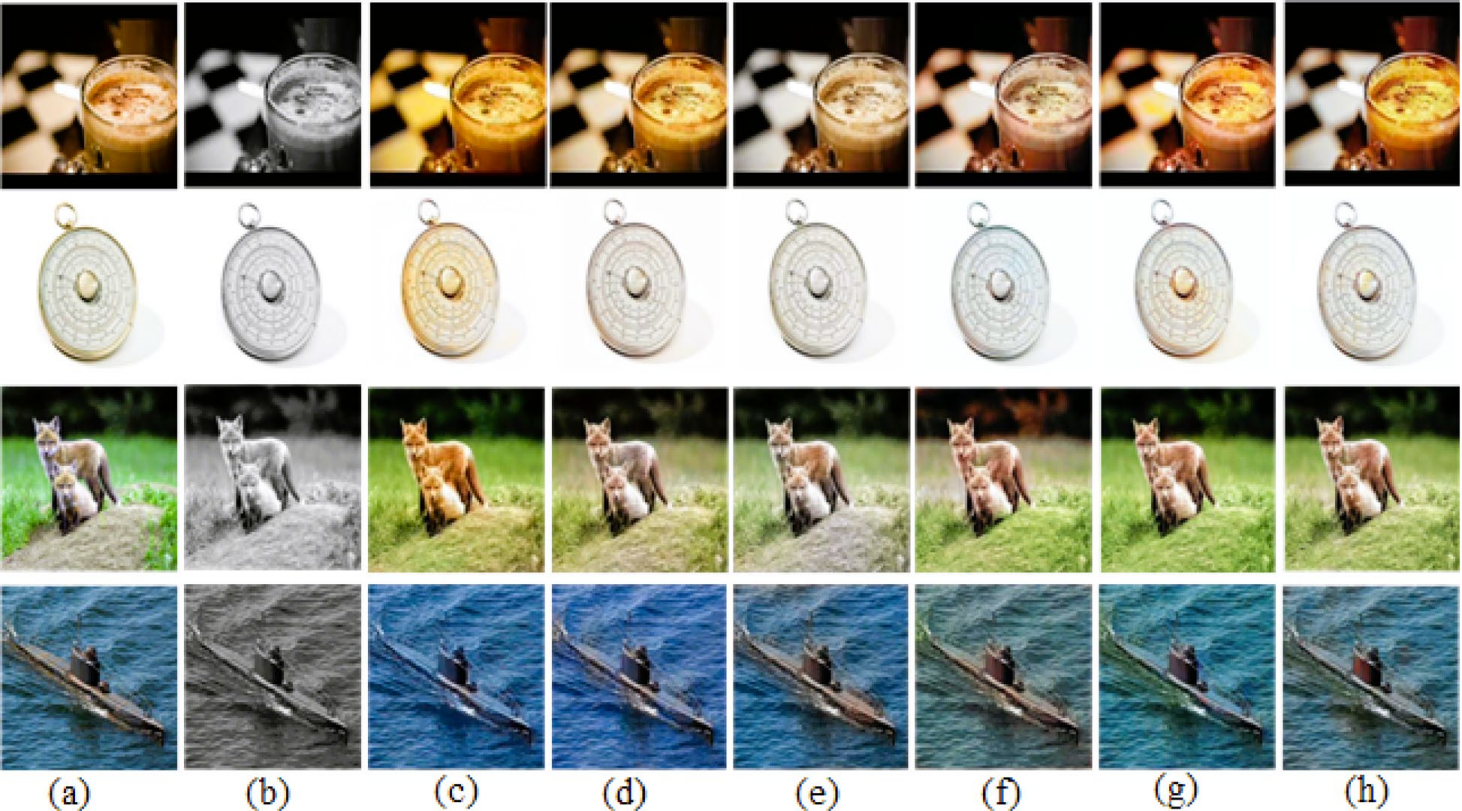
Successful cases of the ImageNet dataset. (**a**) Ground-truth. (**b**) Input image. (**c**) User-guided CNN^8^. (**d**) GAN-based Auto Colorization^9^. (**e**) Transfer Learning with VGG^10^. (**f**) CLIP-based Semantic Guidance^16^. (**g**) Transfer learning with vision transformer^18^. (**h**) Our results with VGG19 + CLAHE.

For the ImageNet^24^ dataset, 500 images from validation set are considered for evaluation. While some existing methods produced reasonable results, our proposed approach achieved the most successful cases, generating outputs that closely resemble the original images, as illustrated in Fig. 4.

**Fig. 5:**
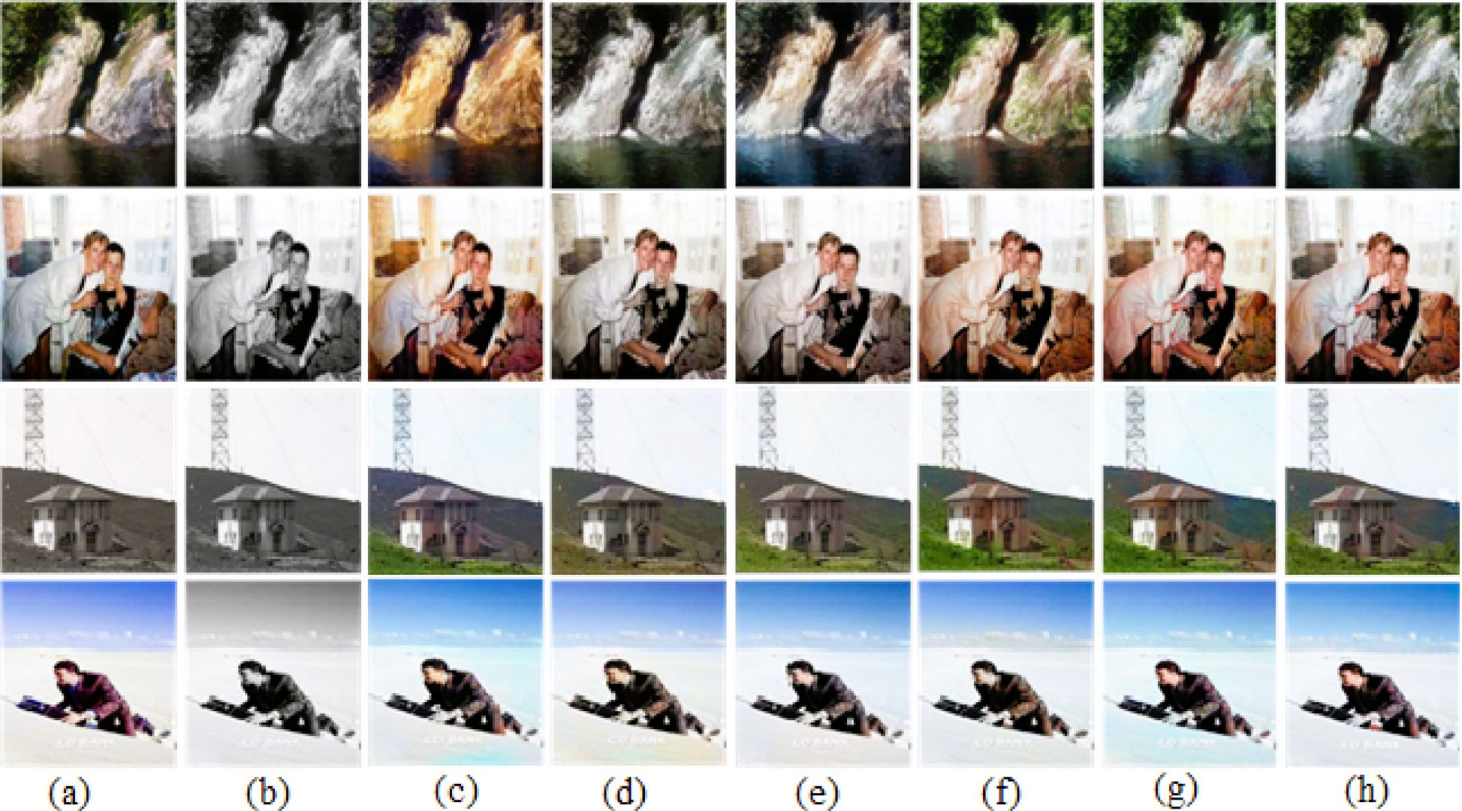
Successful cases of the Places365 dataset. (**a**) Ground-truth. (**b**) Input image. (**c**) User-guided CNN^8^. (**d**) GAN-based auto colorization^9^. (**e**) Transfer learning with VGG^10^. (**f**) CLIP-based semantic guidance^16^. (**g**) Transfer learning with vision transformer^18^. (**h**) Our results with VGG19 + CLAHE.

**Fig. 6:**
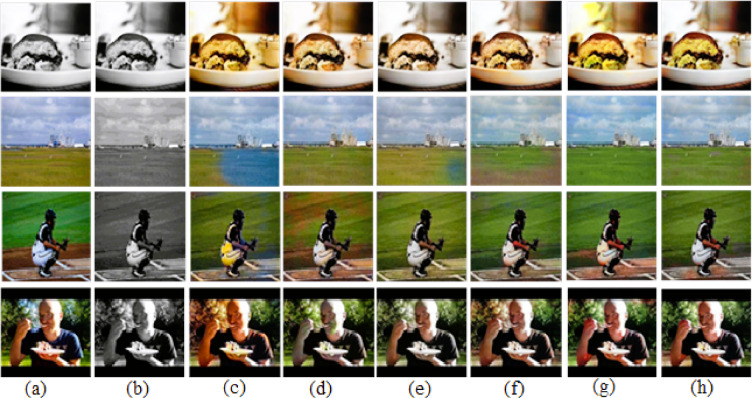
Successful cases of the COCO-Stuff dataset. (**a**) Ground-truth. (**b**) Input image. (**c**) User-guided CNN^8^. (**d**) GAN-based auto colorization^9^. (**e**) Transfer learning with VGG^10^. (**f**) CLIP-based semantic guidance^16^. (**g**) Transfer learning with vision transformer^18^. (**h**) Our results with VGG19 + CLAHE.

**Fig. 7:**
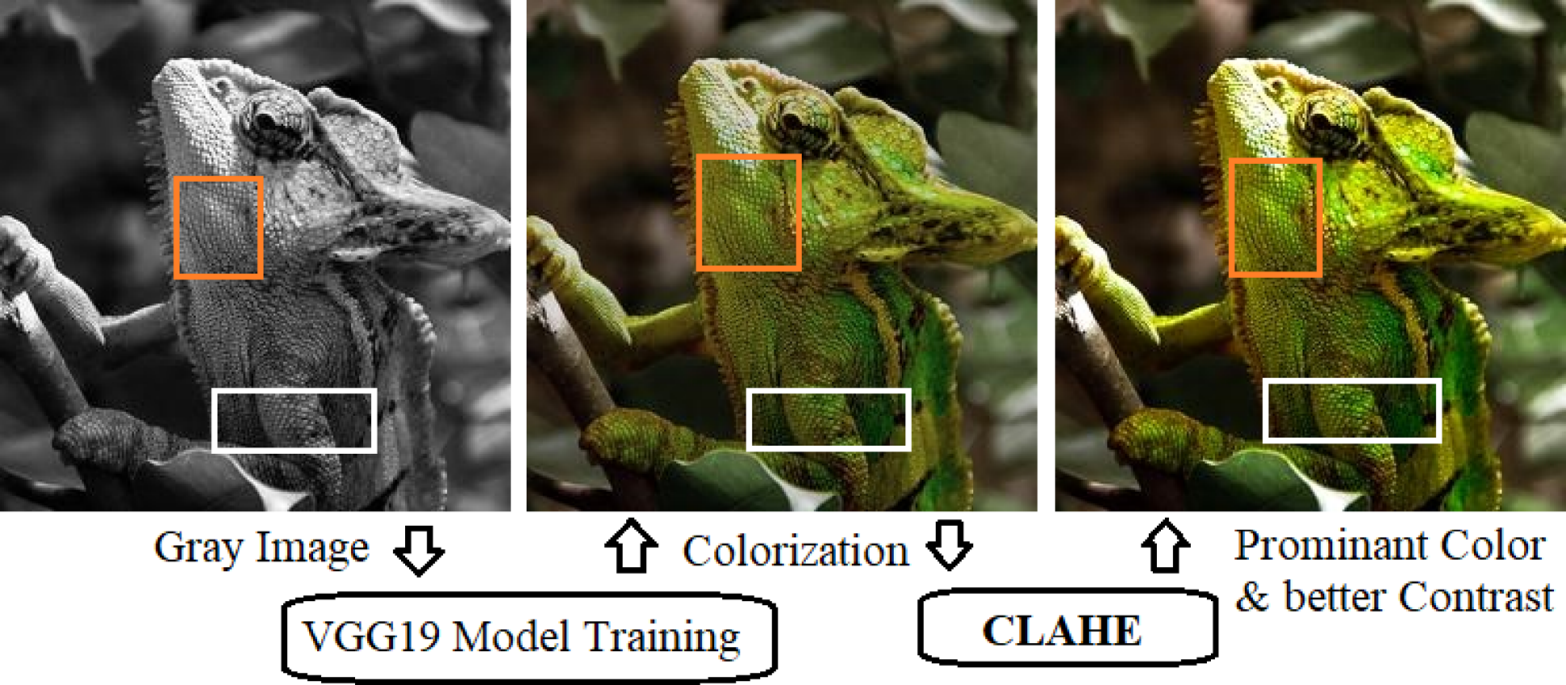
Close view of a successful colorization cases from ImageNet dataset.

We also attempted coloring on another 500 images from Places365^25^ dataset and 500 images from COCO-Stuff^28^ dataset for validation. Overall, our results’ colors were sufficient to create some striking images. Few successful colorization cases of Places365^25^ dataset and COCO-Stuff^28^ dataset are shown in Figs. 5 and 6 respectively. A close view of a successful case is shown in Fig. 7 for better visualization of colorization in detail.

**Fig. 8:**
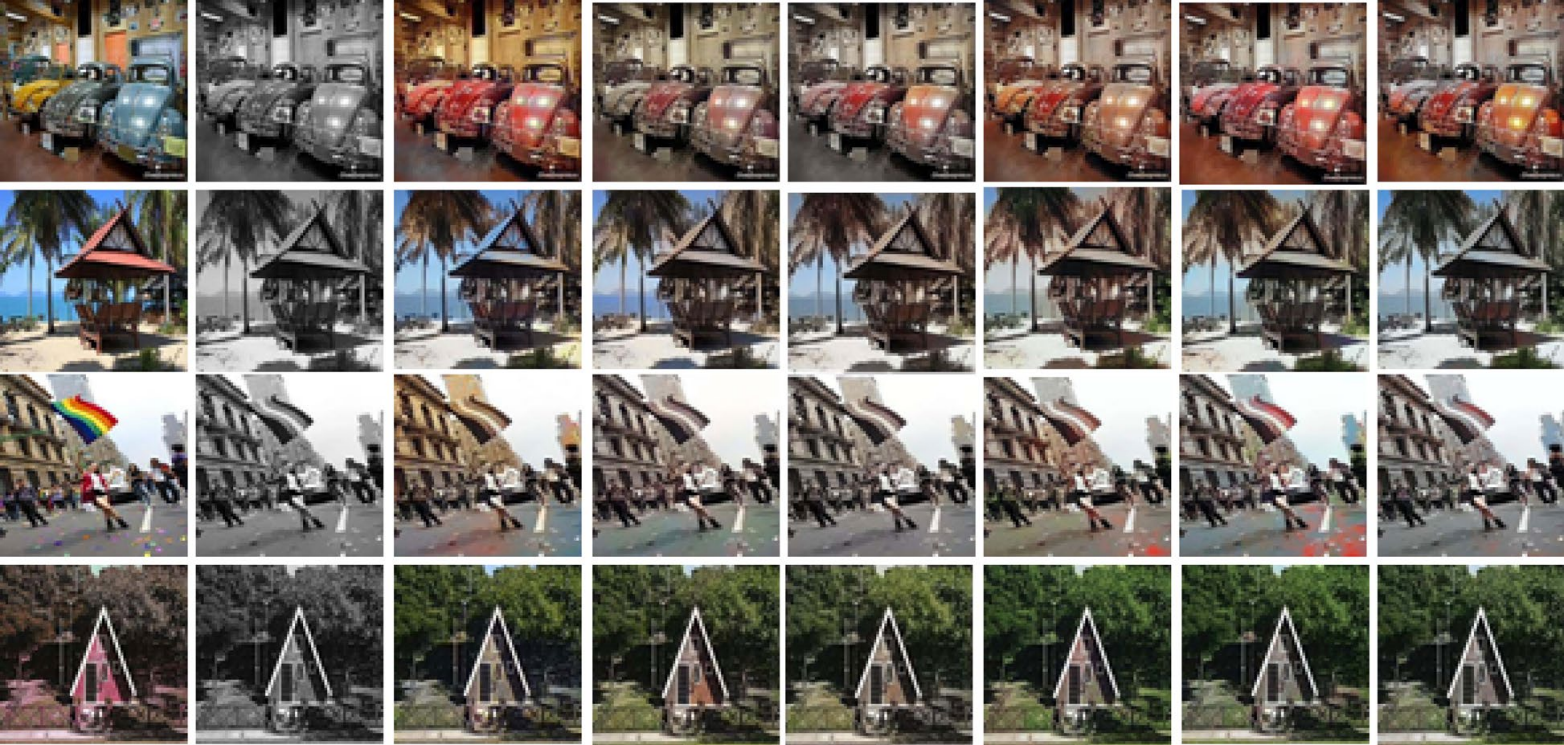
Failure cases where the output images by our proposed method is unable to produce perfect output image exactly like original ground-truth. (**a**) Ground-truth, (**b**) Input image, (**c**) User-guided CNN^8^, (**d**) GAN-based Auto Colorization^9^, (**e**) Transfer Learning with VGG^10^, (**f**) CLIP-based Semantic Guidance^16^, (**g**) Transfer Learning with Vision Transformer^18^, (**h**) Our results with VGG19+CLAHE.

However, the ground-truth images often contain fine-grained details, which present significant challenges for automatic colorization, both for our method and others. In our experiments, we observed instances of inaccurate color assignments and noise, which were categorized as failure cases. Here, the output images by our proposed method are unable to produce a perfect output image exactly like the original ground-truth. Some of the out images were unable to produce exact color. Some of our images produced over-colored images with over-saturation of color. These over saturation and over-colored output images are not as natural as the original ground-truth images. Some of the failure cases are able to produce a closer color like ground-truth, but their luminance is different. As a result, some pixels of the output image look darker or brighter in some cases. A few of the observed failure cases where output images are not natural as ground-truth are shown in Fig. 8.

## Limitation

Although, the proposed hybrid method is well efficient for colorization of gray image, it has some limitations. If, gray value or intensity values are not properly distributed in input image, then colorization will not be perfect. If input image consist of very high intensity across the image in most of the pixel then, the CLAHE will not work properly. Sometimes, the output color produced by proposed methods are not realistic and not looking natural. The colorization will not work properly in case of very dark and very bright input image.

## Conclusion and future scope

This paper presented a deep transfer learning–based image colorization framework using VGG19 and CLAHE within an encoder–decoder architecture. By integrating global semantics from Places365^24^ and local object–scene features from the COCO-Stuff^28^ dataset, the model achieved improved pixel-level colorization through regression and soft-encoding branches. Experiments on ImageNet^24^, COCO-Stuff^28^ and Places365^25^ datasets confirmed its effectiveness, yielding strong results in PSNR, RMSE, SSIM, and Pearson correlation. The study highlights the benefits of multi-skip connections, YUV color space, and small convolutional kernels in reducing artifacts and enhancing feature learning. The method is highly effective for restoring old historical photos and for coloring gray medical scan images. While the model demonstrates robust performance, challenges remain with complex textures, rare colors, and highly cluttered images, leading to occasional noise, mis-colorization, or inconsistency.

Future work will emphasize refining the color distribution model using multi-scale optimization and employing generative models for more realistic and diverse results. Experiments on larger and more diverse datasets will improve generalization across domains. Further exploration of alternative color spaces such as HSV, YCbCr, and CIE Lab may enhance performance. Additionally, deeper U-Net variants and optimized training strategies on high-performance hardware can accelerate learning and improve accuracy. These directions aim to overcome current limitations and advance the reliability of automatic image colorization in real-world applications.

## Data Availability

The datasets used and/or analyzed during the current study are available from the corresponding author on reasonable request.
